# Worry About Caregiving Performance: A Confirmatory Factor Analysis

**DOI:** 10.3389/fmed.2018.00079

**Published:** 2018-03-22

**Authors:** Ruijie Li, Mei Sian Chong, Peng Chew Mark Chan, Bee Gek Laura Tay, Noorhazlina Binte Ali, Wee Shiong Lim

**Affiliations:** ^1^Health Services and Outcomes Research, National Healthcare Group, Singapore, Singapore; ^2^Biostatistics and Health Informatics Department, Institute of Psychiatry, Psychology and Neuroscience, King’s College London, London, United Kingdom; ^3^Geriatric Education and Research Institute, Singapore, Singapore; ^4^Cognition and Memory Disorders Service, Department of Geriatric Medicine, Tan Tock Seng Hospital, Singapore, Singapore; ^5^Institute of Geriatrics and Active Ageing, National Healthcare Group, Singapore, Singapore; ^6^Sengkang Hospital, Singapore, Singapore

**Keywords:** Zarit Burden Interview, caregivers, dementia, dimensions, factor analysis

## Abstract

Recent studies on the Zarit Burden Interview (ZBI) support the existence of a unique factor, worry about caregiving performance (WaP), beyond role and personal strain. Our current study aims to confirm the existence of WaP within the multidimensionality of ZBI and to determine if predictors of WaP differ from the role and personal strain. We performed confirmatory factor analysis (CFA) on 466 caregiver-patient dyads to compare between one-factor (total score), two-factor (role/personal strain), three-factor (role/personal strain and WaP), and four-factor models (role strain split into two factors). We conducted linear regression analyses to explore the relationships between different ZBI factors with socio-demographic and disease characteristics, and investigated the stage-dependent differences between WaP with role and personal strain by dyadic relationship. The four-factor structure that incorporated WaP and split role strain into two factors yielded the best fit. Linear regression analyses reveal that different variables significantly predict WaP (adult child caregiver and Neuropsychiatric Inventory Questionnaire (NPI-Q) severity) from role/personal strain (adult child caregiver, instrumental activities of daily living, and NPI-Q distress). Unlike other factors, WaP was significantly endorsed in early cognitive impairment. Among spouses, WaP remained low across Clinical Dementia Rating (CDR) stages until a sharp rise in CDR 3; adult child and sibling caregivers experience a gradual rise throughout the stages. Our results affirm the existence of WaP as a unique factor. Future research should explore the potential of WaP as a possible intervention target to improve self-efficacy in the milder stages of burden.

## Introduction

Dementia is a disease that is frequently associated with significant caregiving burden. One of the most widely used instruments to quantify caregiving burden is the Zarit Burden Interview (ZBI) (1). The ZBI has been validated in different populations and has been shown to be invariant across different educational levels and gender (2). The degree of caregiving burden has traditionally been assessed using pre-defined cutoffs on the ZBI total score, essentially constituting a unidimensional approach (3, 4). Subsequent studies have since pointed toward caregiving burden as a multidimensional construct. The seminal study by Whitlatch and colleagues was the first to outline the dual-factor structure of role and personal strain as distinct constructs measured by the ZBI (5). Role strain refers to how the caregiving role is in conflict with other roles that the caregiver has to manage while personal strain refers to how the caregiving experience is personally stressful. Subsequent studies have built upon the general structure of role and personal strain and partially replicated the factor structure (6–10). This partial replication across diverse populations with cultural and societal differences (11) raises the possibility of a latent dimension beyond the general structure of role and personal strain.

Of note, a factor has consistently emerged in recent studies, known variously as: “self-criticism” (9, 10, 12), “guilt”(13–15), “feelings of inadequacy” (16, 17), and “worry about performance” (7, 8). This factor highlights a distinct dimension of burden describing caregiver concerns about doing more (item 20) and doing a better job (item 21), either in isolation or in combination with other items (3). It represents a conceptual continuum of a negative aspect of caregiving arising from self-appraisal of caregiving performance (18), ranging from milder degrees of “inadequacy” and “worry” to “self-criticism” and “guilt” on the severe end. The low correlation with other factors and total ZBI score, consistency of items 20 and 21 co-occurring in same factor, and its conceptual consistency across the continuum of self-appraisal of caregiving performance corroborate the existence of this unique construct within the ZBI.

This provided the basis for our earlier proposal that there are three key dimensions that underpin ZBI-defined burden, namely role strain, personal strain, and the unique factor comprising items 20 and 21, which we termed worry about caregiving performance (WaP) (3). Using exploratory factor analysis in a multiethnic Chinese predominant Asian context, we demonstrated the presence of the unique factor WaP above and beyond role and personal strain (8). In addition, our factor solutions outlined two possible components of “role strain” comprising “role strain (demands)” and “role strain (control).” These findings are consistent with the broader literature that reports the multidimensionality of ZBI beyond the dual-factor structure, which was originally proposed by Whitlatch and colleagues (5). The number of ZBI factors reported in these studies ranged from three to five, suggesting that additional factors beyond the three core components may represent variants of either role or personal strain. In support of this, a recent study in an Asian Chinese population similarly reported an optimal four-factor structure comprising two factors of role strain (captivity and loss of control), and one factor each of personal strain and self-criticism (items 20 and 21) (9).

The relationship between WaP with various socio-demographic and diseases characteristics is hitherto not well understood. Unlike role strain and personal strain, stressors of functional impairment and neuropsychiatric symptoms do not predict WaP (3, 8, 13). Previous studies reported younger age of caregiver as a major predictor of WaP and a significant elevation of scores in the mild stage (3, 8, 13). This suggests that the inverse relationship of age with WaP is indicative of higher levels of WaP burden among adult children relative to spousal and other caregivers (19). The influence of relationship with care recipient on the variation of WaP across the severity spectrum of cognitive impairment remains to be elucidated (8). Earlier studies also focused on the impact of severity of neuropsychiatric symptoms rather than the resultant distress from these symptoms (3, 13).

This provided the impetus for our current follow-up study in a separate cohort of predominantly Chinese multiethnic Asian population attending a memory clinic. Our primary objective was to determine if WaP is a unique factor that exists within the ZBI, and whether splitting role strain into two factors contributes to a better model fit as opposed to keeping it intact as one factor. The secondary objective was to explore the relationships of the various factors of ZBI in relation to socio-demographic variables and disease characteristics, and how relationship with care recipient (adult children, spouse and sibling) can influence the factor scores across the severity spectrum of cognitive impairment.

## Materials and Methods

### Study Design and Participants

This is a cross-sectional study of 466 caregiver-patient dyads of community-dwelling older adults with cognitive complaints presenting for the first time to the Memory Clinic, Tan Tock Seng Hospital, Singapore, from January 2010 to December 2011. The study was approved by the institutional review board of the National Healthcare Group.

We included caregiver-patient dyads who fulfilled the following criteria: (1) patients who were aged 55 years and above with a Clinical Dementia Rating (CDR) (20) global score of >0 and a diagnosis of mild cognitive impairment (MCI) or dementia; (2) community-dwelling patients who were not residing in an assisted living facility or nursing home; (3) primary caregiver of the patient, defined as the family member above 21 years of age who was most involved in the provision of day-to-day care and who was familiar with the patient’s medical and social condition. We excluded the following categories of caregivers: (1) non-family members (e.g., domestic helper, friend); (2) inability to converse fluently in English or Mandarin; (3) refusal to fill out the ZBI. Among 784 caregiver-patient dyads presenting for the first time to the memory clinic over the 2-year period, 466 caregiver-patient dyads were recruited based on the inclusion and exclusion criteria.

### Assessment

Details of the evaluative approach at the memory clinic have been previously described (21). All MCI and dementia subjects in this study underwent detailed semi-structured clinical evaluation, as well as relevant laboratory investigations and neuroimaging to exclude potentially reversible causes of cognitive impairment. Standardized neuropsychological assessment was performed on all MCI subjects. A consensus meeting was conducted to determine the diagnosis, etiology, and staging of cognitive impairment based on inputs from the multi-disciplinary team comprising physicians, nurse clinicians, and psychologists.

The severity of cognitive impairment was staged using the CDR (20). A CDR of 0 indicates no cognitive impairment; 0.5 indicates either MCI or very mild dementia; and 1, 2, and 3 indicate mild, moderate, and severe dementia, respectively. Convergent validity of the CDR to discriminate milder stages of dementia has been demonstrated locally (22). Our operational definition of the MCI subgroup in accordance with the International Working Group criteria has been previously described (21). Dementia was diagnosed according to the Diagnostic and Statistical Manual of Mental Disorders, Fourth Edition, Text Revision (23). The dementia etiologic subgroups of Alzheimer’s disease (AD), vascular dementia (VD) and mixed dementia were made using standardized criteria such as National Institute of Neurological and Communicative Disorders & Stroke—Alzheimer’s Disease and Related Disorders Association (NINCDS-ADRDA) (24) and National Institute of Neurological Disorders and Stroke—Association Internationale pour la Recherche et l’Enseignement en Neurosciences (NINDS-AIREN) (25).

### Measurements and Instruments

We collected socio-demographic characteristics of the patients and caregivers such as age, gender, ethnicity, level of education, family relationship (spouse, adult children, sibling, or others), and living situation (with or apart from patient). Cognition was assessed using the Chinese Mini Mental Status Examination (CMMSE), which has been validated locally (26). This version has modifications made to the original instrument to ensure its relevance locally. This modified instrument has a total score of 28 with lower scores indicating lower cognitive abilities.

Functional assessment consisted of the Modified Barthel Index (MBI) (27) and the Lawton scale (28). The MBI measures the degree of independence in 10 self-care tasks. It is scored 0 to 100 with a higher score indicating greater independence in basic activities of daily living (BADL). The Lawton scale measures the degree of independence in more complex instrumental activities of daily living (IADL) such as housekeeping, shopping, handling finances, and meal preparation. Patients were scored 0 to 23 with higher scores indicating greater independence.

Neuropsychiatric symptoms were assessed using the Neuropsychiatric Inventory Questionnaire (NPI-Q) (29). The two components of the NPI-Q, severity and distress, were scored 0 to 3 and 0 to 5, respectively. Severity is an indication of seriousness and distress is an indication of the stress experienced by the caregiver for a symptom or area of concern. Both the severity and distress scores were used as they reflect different perspectives in relation to caregiving burden from the patient and caregiver, respectively.

Caregiver burden was measured using the Zarit Caregiver Burden (ZBI), which is a self-administered 22-item instrument for caregivers (1). The questions are scored on a 5-point Likert scale ranging from 0 to 4 corresponding to “never,” “rarely,” “sometimes,” “quite frequently,” and “nearly always.” Individual items are summated to yield a maximum possible score of 88, with higher scores indicating greater burden. For caregivers who were unable to comprehend the English version, a validated Chinese version was used instead (30). The validation of the Chinese version included back-translation procedures which ensured that both versions are being interpreted similarly. This mitigates concerns of the threats to internal validity in the use of both languages.

### Statistical Analysis

We made comparisons of model fits between five different factor models of ZBI derived from literature and previous work, such as (1) unidimensional model (all items); (2) two-factor model comprising role strain (items 1, 4, 5, 8, 9, 14, 16, 17, 18, 19, 20, and 21) and personal strain (items 2, 3, 6, 11, 12, and 13) (5); (3) three-factor model adapted from Cheah and colleagues (8), comprising a single role strain factor (items 1, 2, 3, 4, 7, 8, 11, 12, 14, 13, 15, 16, 17, and 18), a personal strain factor (items 5, 6, 9, 10, 19, and 22) and WaP (items 20, 21); (4) four-factor model following the original four factors of Cheah and colleagues (8), namely role strain due to demands of care (items 1, 2, 3, 4, 7, 8, 11, 12, and 14), role strain secondary to loss of control over the situation (items 13, 15, 16, 17, and 18), personal strain and worry about performance and (5) four-factor model by Cheng and colleagues (9) comprising captivity (items 11, 12, 13 and 14), loss of control (items 16, 17 and 19), personal strain (items 1, 2, 3, 4, 6, 7, 8, 9 and 10), and self-criticism (items 20 and 21).

We included item 22 (“Overall, how burdened do you feel in caring for your relative?”) in the analysis. Several prior studies opted not to include this item (9, 12, 15, 17, 31, 32) while others did (6–8, 13, 33, 34). The studies that included item 22 cited its global nature and high correlation with other factors of the ZBI as a reason to exclude it from analysis. While item 22 theoretically represents an overall perception of burden by the caregivers, caregivers may interpret it differently and align their answer closer to one of the latent factors. This is evident from its loading on the personal strain factor in our prior study (8). To be conceptually aligned with these prior results, we elected to retain item 22 in the analyses.

We conducted confirmatory factor analysis (CFA) to determine if the presence of WaP (models 3–5) improves the model fit compared with total ZBI score (model 1) and role/personal strain (model 2). CFA was also used to assess if splitting role strain into two factors (models 4 and 5) is superior to retaining it as one factor (model 3). Robust weighted least squares were used as the estimator for the CFA as the ZBI is an ordinal scale. We used different indices to compare the fit of the four models: χ^2^, root mean square error of approximation (RMSEA), standardized root mean square residual (SRMR), non-normed fit index (NNFI), and comparative fit index (CFI). We used the criteria proposed by Hu and Bentler (35) to determine a good fit, namely RMSEA (<0.06), SRMR (<0.08), NNFI (>0.95), and CFI (>0.95). In addition, we compared all the models against the one-factor model using χ^2^ difference tests as the one-factor model is nested within all models with more than one factor.

From the best fitting model, factor scores for each factor were computed for use in the subsequent analyses. We computed correlations between the factors to determine how they relate to each other. We also performed multiple linear regression analyses to explore the relationships between ZBI total and factor scores with the candidate predictor variables MBI, Lawton IADL, NPI-Q severity, NPI-Q distress, and CMMSE. These variables were chosen based on previous work (3) with three modifications. First, we included both scales of the NPI-Q to better understand the differential impact of symptom severity versus resultant distress on caregiver burden. Second, we included the CDR stage of the patient to determine if disease severity had any bearing on the level of burden on the caregivers. Third, we substituted caregiver age with “relationship with patient” due to strong collinearity between these two variables, assessed using generalized variance inflation factors and correlation matrices. We chose to include “relationship with patient” in the final model as we wanted to ascertain whether our previous finding of a relationship between younger age and caregiving burden could be explained by different dyadic relationship with the care recipient (3). To further clarify this relationship, we investigated the profile of each factor score across CDR score ranges, stratified by dyadic relationship. CDR was used as it is a uniform gauge of the severity of dementia in general (36).

All analyses were conducted using R 3.1.2 and Mplus 7. Descriptive statistics were used to describe our sample. Mean and standard deviation values were computed for continuous variables, and frequencies were computed for categorical variables. For inferential statistics, the *p*-value threshold considered significant was set at 0.05. Missing data and some socio-demographic variables were present in the ZBI. For the ZBI, we imputed the seven cases with one missing data point per case with the median of each question. The median was used to retain the ordinal nature and interpretability of the scale used in the ZBI. For socio-demographic variables, the 63 cases with missing data were not significantly different in all measures reported in the study (*p* > 0.05) and hence were excluded from regression analyses.

## Results

### Characteristics of Caregivers and Patients

Four-hundred sixty-six caregiver-patients dyads were included in the study (Table 1). Caregivers had a mean age of 53.8 years (SD = 13.5) and were predominantly Chinese female with an average of 11 years of formal education (SD = 4.5). Most of the caregivers were adult children (61.4%) followed by spouses (26.6%). The patients had a mean age of 76.4 years (SD = 7.4), were predominantly female Chinese with an average of 4.9 years of formal education (SD = 4.7). Compared with adult children caregivers, sibling caregivers tended to be older [age, mean (SD): 47.43 (8.70) vs. 64.63 (9.07)] and mainly females (65.73 vs. 87.50%). Most of the patients were diagnosed with AD (53.2%) followed by other forms of dementia (i.e., not VD or mixed dementia) (21.1%). The majority (44.2%) had mild dementia (CDR 1.0), followed by moderate dementia (CDR 2.0) (27.3%). The mean total ZBI score was 24.9 (SD = 17.4), and the mean factor z-scores were −0.003 (SD = 0.978), −0.008 (SD = 0.976), 0.002 (SD = 0.957), and 0.024 (SD = 0.859) for personal strain, role strain (control), role strain (demands), and WaP, respectively.

**Table 1:** Sample characteristics (*n* = 466).

Patient characteristics	
Age in years	76.4 (7.4)
Female gender, *n* (%)	275 (59.0)
Education level in years	4.9 (4.7)
Ethnic Group, *n* (%)	
Chinese	417 (89.5)
Malay	14 (3.0)
Indian	29 (6.2)
Others	6 (1.3)
**Disease characteristics**	
Global CDR score, *n* (%)	
CDR 0.5 (MCI)	58 (12.4)
CDR 0.5 (very mild dementia)	60 (12.9)
CDR 1.0 (mild dementia)	206 (44.2)
CDR 2.0 (moderate dementia)	127 (27.3)
CDR 3.0 (severe dementia)	15 (3.2)
Dementia types, *n* (%)^a^	
AD	217 (53.2)
VD	79 (19.4)
Mixed AD/VD	26 (6.4)
Others	86 (21.1)
CMMSE (range 0–28)	16.6 (6.1)
BADL (range 0–100)	92.9 (36.4)
IADL (range 0–23)	12.2 (5.9)
NPI-Q	
Severity (range 0–36)	5.6 (5.0)
Distress (range 0–60)	5.9 (7.3)
**Caregiver characteristics**	
Age in years	53.8 (13.5)
Female gender, *n* (%)	287 (61.6)
Education level in years	11 (4.5)
Relationship with patient, *n* (%)	
Spouse	124 (26.6)
Adult children	286 (61.4)
Sibling	8 (1.7)
Others	48 (10.3)
Living with patient, *n* (%)	351 (75.3)
ZBI score (range 0–88)	24.9 (17.4)

*^a^n = 408, excluding MCI cases*.

*Mean (SD) unless otherwise stated*.

*AD, Alzheimer’s dementia; BADL, Basic Activities of Daily Living; CDR, Clinical Dementia Rating; CMMSE, Chinese Mini Mental Status Examination; IADL, Instrumental Activities of Daily Living; MCI, mild cognitive impairment; NPI-Q, Neuropsychiatric Inventory Questionnaire; VD, Vascular dementia; ZBI, Zarit Burden Interview*.

### CFA of ZBI

The χ^2^ difference tests were all significant, suggesting that models with more than one factor fit the data better than the one-factor model. Table 2 shows the fit indices used to determine the best fit among the four factor models. RMSEA, NNFI, and CFI do not fit the criteria for a good fit for all five models. For SRMR, all the models fit the criteria for a good fit with the exception of the one-factor model. In making the comparison to determine if the presence of WaP improved model fit, we noted that NNFI for factor models without WaP was lower than models with WaP. This is of particular significance as NNFI penalizes models for greater complexity and the factor models with WaP are more complex than the factor models without WaP.

**Table 2:** CFA fit indices.

	*df*	χ^2^		RMSEA	SRMR	NNFI	CFI
1 factor (Zarit and Zarit, 1982) (37)	209	1849.888	***	0.130	0.080	0.863	0.876
2 factor (Whitlatch et al., 1991) (5)	134	1543.840	***	0.150	0.087	0.844	0.864
3 factor (Cheah et al., 2012) (8)	206	1018.985	***	0.092	0.065	0.931	0.939
4 factor (Cheah et al., 2012) (8)	203	969.183	***	0.090	0.063	0.934	0.942
4 factor (Cheng et al., 2014) (9)	129	689.290	***	0.097	0.061	0.938	0.948

*p < 0.05; **p < 0.01; ***p < 0.001.

*CFA, confirmatory factor analysis; CFI, Comparative Fit Index; df, degrees of freedom; NNFI, Non-normed Fit Index; RMSEA, Root Mean Square Error of Approximation; SRMR, Standardized Root Mean Square Residual*.

We also compared the three- and four-factor models from our previous work to determine if splitting role strain into two factors is better than keeping it as one. The fit indices unanimously indicate that the four-factor models were superior in fit to the three-factor model although the differences were quite small. In view of the better explanatory power of the four-factor model, we opted to use the four-factor model for further analyses. Table 3 shows the standardized factor loadings and standard errors of the four-factor model.

**Table 3:** Standardized factor loadings and standard errors for the four-factor Zarit Burden Interview model (8).

Items	Role strain (control)	Role strain (demands)	Personal strain	Worry about caregiving performance	SE
I01	0.587				0.032
I02	0.822				0.017
I03	0.834				0.016
I04	0.640				0.033
I07	0.611				0.030
I08	0.718				0.026
I11	0.846				0.017
I12	0.859				0.016
I14	0.656				0.029
I13		0.779			0.028
I15		0.660			0.031
I16		0.835			0.021
I17		0.881			0.018
I18		0.796			0.023
I05			0.709		0.027
I06			0.733		0.026
I09			0.826		0.017
I10			0.811		0.021
I19			0.716		0.026
I22			0.852		0.016
I20				0.957	0.028
I21				0.807	0.029

### Correlations

Correlation analysis was performed using the four factors as reported by Cheah and colleagues (8) (Table 4). WaP correlated only moderately with the other three factors (*r* = 0.572–0.588, *p* < 0.001) and total ZBI (*r* = 0.624, *p* < 0.001), unlike the high correlation seen among the other factor scores (*r* = 0.956–0.991, *p* < 0.001).

**Table 4:** Correlation matrix between factors and total ZBI score and Cronbach’s α.

	Role strain (demands)	Role strain (control)	Personal strain	Worry about performance	ZBI total
Role strain (demands)		***	***	***	***
Role strain (control)	0.966		***	***	***
Personal strain	0.990	0.991		***	***
Worry about performance	0.572	0.588	0.587		***
ZBI total	0.956	0.960	0.966	0.624	

*p < 0.05; **p < 0.01; ***p < 0.001.

*ZBI, Zarit Burden Interview*.

### Predictors of Different Factors of ZBI

Linear regressions conducted on ZBI total and factor scores were all significant (*p* < 0.05), with the WaP model having a much lower adjusted *R*^2^ (0.091) compared to the other models (0.251–0.283) (Table 5). Significant predictors for total ZBI, role strain (demands), role strain (confidence), and personal strain are relationship (adult child), IADL and NPI-Q distress, compared with relationship (adult child) and NPI-Q severity for WaP. Notably, IADL and NPI-Q distress were significantly associated with all factors of the ZBI except for WaP; the reverse was true for NPI-Q severity. BADL, CMMSE, caregiver gender, co-residence, and severity of dementia (relative to MCI as reference group) did not predict total ZBI or factor scores.

**Table 5:** Regression of factors and ZBI total score on caregiver and care recipient characteristics.

	Total ZBI				Role strain (demands)				Role strain (control)				Personal strain				Worry about performance			
Regression	*b*	β	*p*-Value		*b*	β	*p*-Value		*b*	β	*p*-Value		*b*	β	*p*-Value		*b*	β	*p*-Value	
**Relationship with care recipient (reference: spouse)**																				
Adult child	0.207	0.126	0.023	*	0.245	0.123	0.029	*	0.316	0.161	0.005	**	0.284	0.142	0.012	*	0.329	0.184	0.003	**
Sibling	0.509	0.092	0.048	*	0.506	0.075	0.112		0.693	0.104	0.029	*	0.631	0.093	0.049	*	−0.062	−0.010	0.845	
Others	0.085	0.016	0.726		0.086	0.014	0.772		0.032	0.005	0.915		0.053	0.008	0.860		0.172	0.030	0.562	
**Caregiver gender (reference: female)**																				
Male	−0.007	−0.004	0.918		−0.032	−0.016	0.716		0.002	0.001	0.986		−0.014	−0.007	0.874		−0.081	−0.045	0.359	
**Living with care recipient (reference: no)**																				
Yes	0.022	0.012	0.796		0.039	0.017	0.714		0.025	0.011	0.812		0.025	0.011	0.813		−0.151	−0.071	0.158	
Caregiver education	−0.010	−0.061	0.244		−0.008	−0.039	0.457		−0.016	−0.080	0.134		−0.012	−0.060	0.258		−0.002	−0.011	0.853	
BADL (0−100)	−0.001	−0.026	0.581		−0.001	−0.023	0.638		0.000	−0.013	0.792		0.000	−0.013	0.794		0.001	0.057	0.288	
IADL (0−23)	−0.017	−0.129	0.031	*	−0.028	−0.175	0.004	**	−0.025	−0.156	0.011	*	−0.025	−0.159	0.009	**	0.003	0.021	0.756	
NPI-Q severity (0–36)	0.011	0.071	0.407		0.007	0.040	0.647		0.014	0.077	0.375		0.010	0.055	0.522		0.032	0.189	0.049	*
NPI-Q distress (0–60)	0.035	0.336	0.000	***	0.042	0.323	0.000	***	0.037	0.289	0.001	***	0.041	0.321	0.000	***	0.001	0.009	0.924	
CMMSE (0–28)	0.004	0.030	0.635		0.006	0.037	0.565		0.005	0.034	0.606		0.006	0.037	0.567		−0.002	−0.011	0.875	
**CDR [Reference: CDR 0.5 (MCI)]**																				
CDR 0.5 (very mild dementia)	−0.081	−0.035	0.550		−0.121	−0.043	0.469		−0.076	−0.027	0.650		−0.099	−0.035	0.557		0.240	0.095	0.151	
CDR 1.0 (mild dementia)	0.060	0.039	0.610		0.059	0.031	0.686		0.081	0.044	0.576		0.069	0.037	0.637		0.160	0.094	0.272	
CDR 2.0 (moderate dementia)	0.198	0.114	0.205		0.198	0.094	0.304		0.172	0.083	0.370		0.185	0.088	0.340		0.326	0.173	0.090	
CDR 3.0 (severe dementia)	0.438	0.092	0.114		0.541	0.094	0.114		0.418	0.074	0.220		0.514	0.089	0.136		0.441	0.085	0.196	
Adjusted *R*^2^	0.285		0.000	***	0.262		0.000	***	0.248		0.000	***	0.260		000	***	0.091		0.000	***

*p < 0.05; **p < 0.01; ***p < 0.001.

*BADL, basic activities of daily living; IADL, Instrumental Activities of Daily Living; MCI, mild cognitive impairment; NPI-Q, Neuropsychiatric Inventory Questionnaire*.

### Relationship Across Disease Severity by Different Dyadic Relationships

**Figure 1:**
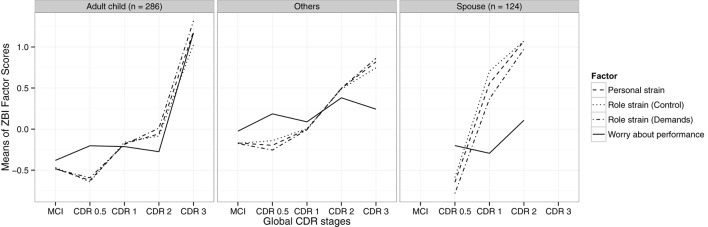
Zarit Burden Interview (ZBI) total and factor scores by disease severity of different dyadic relationships.

WaP exhibits a unique trajectory across the CDR stages by different dyadic relationships when compared with the other three factors (Figure 1). A limitation of the plot is the lack of data points for MCI and CDR 3 for the “sibling as caregiver” plot. In all three dyadic relationships, WaP had the highest score in MCI and CDR 0.5 dementia. Among adult child and sibling caregivers, WaP showed only a modest increase moving across the CDR stages, in contrast to the much steeper increase with increasing dementia severity for the other three factors. Among spousal caregivers, WaP remains relatively stable from MCI to CDR 2, unlike the general trend of increase for the other factors; all four factors exhibit a corresponding steep rise moving from CDR 2 to 3 stages.

## Discussion

Our study adds to the growing body of evidence that supports the existence of WaP as a distinct dimension of caregiving burden, and thus corroborates our earlier proposal that the three key dimensions of role strain, personal strain, and WaP underpin the multidimensionality of ZBI-defined burden. It also supports splitting role strain into two factors given the superior model fit. In addition, the regression analysis furthered our understanding of the predictors of WaP by highlighting the differential impact of NPI-Q distress relative to NPI-severity, and affirming the influence of dyadic relationship in previously reported observations of an inverse age relationship with WaP. To our knowledge, this is also the first study to demonstrate how WaP trends differently across the CDR stages for different dyadic relationships compared to the other three factors.

Comparisons of the models within a CFA framework provided the first piece of evidence that WaP is a distinct dimension of caregiving burden. While the majority of fit indices suggest that the data did not have a good fit with the various competing models, there is a consistent trend of superior fit indices in all models that incorporated WaP. Second, the weaker correlation of WaP with total ZBI score and the other factors supports that WaP measures a distinct domain when compared to role and personal strain. Third, in regression analysis, the WaP model has a much lower *R*^2^ and yielded predictors that differ from the more “conventional” predictors of role and personal strain. Fourth, WaP exhibits a distinct trajectory across the different stages of cognitive impairment for different dyadic relationships. Taken together, the above evidence strongly supports the construct validity of WaP as a distinct dimension within ZBI-defined burden.

The CFA also provides evidence that splitting role strain into two factors contributes to a better model fit as opposed to keeping it intact as one factor. While the improvement in fit indices may be slight, earlier studies suggest that the items assigned to the two factors of role strain are qualitatively different (8, 9). One role strain factor assesses the demands of care imposed on the caregiver while the other role strain factor assesses the amount of confidence and control over situations imposed by the caregiving role (8). Thus, both factors relate to distinct aspects of the role of a caregiver, differentiating them from personal strain and WaP factors.

In our regression analyses, we also explicated the discordance in earlier studies regarding the influence of behavioral and psychological symptoms of dementia (BPSD) on WaP (8, 9). Specifically, we found that NPI-Q distress was a significant predictor for both role and personal strain, while NPI-Q severity was a significant predictor for WaP. This difference suggests that different mechanisms possibly drive the different factors of caregiving burden. For role and personal strain, the appraisal of how each neuropsychiatric symptom is distressing possibly relates more to the amount of effort the caregiver has to put in to properly assume the role and manage their own psychological well-being (38). In contrast, for WaP, the caregiver may associate the severity of neuropsychiatric symptoms as a reflection of how well one is performing as a caregiver, thus resulting in burden arising from worry about one’s caregiving performance (3, 18). These results suggest the importance of managing both the severity and distress from BPSD to address different aspects of caregiving burden.

While the difference in general burden levels between the adult child and spouse is well documented (39), this study is the first to investigate the stage-dependent differences between WaP and other factor scores by dyadic relationship. Our results affirm the findings of earlier studies that unlike the other factors, WaP is significantly endorsed even in the milder stages of cognitive impairment (3, 8). Investigation of the interaction between dyadic relationship and stage of cognitive impairment further reveals that WaP increased only slightly in CDR 2–3 among adult children/sibling caregivers, unlike spousal caregivers where WaP remained relatively stable until the steep rise in CDR 3. This suggests a differential pattern of self-appraisal of caregiving performance between the two groups. Among adult children caregivers, WaP may represent in the milder stages of cognitive impairment worry and anxiety about caregiving performance in relation to the strong sense of obligation on assuming the caregiving role, which can progress to more complicated feelings associated with caregiving such as self-criticism and inadequacy if left unaddressed (3, 17). In contrast, among spousal caregivers, the sharp rise of WaP in CDR 3 may herald the onset of guilt in conjunction with overall caregiver stress arising from decompensated coping mechanisms due to increased care demands from functional needs or behavioral disturbances (3, 13). This corresponds to earlier findings that WaP interacts with personal strain to increase total ZBI in higher burden states (3).

Our results raise the intriguing question about the possible relationship between WaP with mastery and self-efficacy beliefs among caregivers. Self-efficacy refers to an individual’s assessment of his or her ability to perform specific activities and achieve a desired outcome (40). Whereas the related concept of mastery refers to a global assessment (41), self-efficacy pertains to beliefs about one’s competence to successfully perform discrete or specific tasks. Self-efficacy beliefs influence the initiation and maintenance of effort in demanding situations, and may vary across specific activities of caregiving such as performing ADLs, handling problem behaviors, and use of community support services (42). Self-efficacy has been found to predict caregiver burden and depressive symptoms (43, 44); it also mediates the influence of social support on caregiver well-being, as well as the response to skill-building psychoeducational intervention programs (45, 46). An understanding of the natural history of WaP and how it interacts with self-efficacy may provide useful insights about potential interventional strategies. For instance, caregivers with milder degrees of WaP burden and low self-efficacy may benefit from specific intervention programs to equip them with the necessary coping skills, thereby increasing the sense of self-efficacy and mastery and averting the slippery slope to more negative appraisals of one’s caregiving performance that may result in guilt, overall caregiver stress, and ultimately burnout (47). Future studies should explore whether WaP, akin to the more established factors of role and personal strain, can be amenable to intervention if detected early (48, 49). This is especially salient in light of the findings that WaP, unlike the other dimensions, occurs early in MCI and CDR 0.5 dementia (8), and in the trajectory of multidimensional ZBI burden (14).

Taken together, our results corroborate WaP as a unique factor that is distinct from role or personal strain. We conceptualize WaP as part of a broader concept of self-appraisal of caregiving performance that encompasses both positive and negative valences (3, 18). Rather than a yes/no dichotomous phenomenon, WaP is likely to represent a continuum that ranges from more positive aspects of conscientious and wanting to do better, through intermediate degrees of inadequacy and self-criticism, to guilt and shame at the negative end of the extreme. However, given that it only has two items, it is inherently an unreliable factor and a decision needs to be made to either remove it from the ZBI or to expand it (9, 50). Taking into account the insights that WaP can confer, especially when viewed in relation to stage of disease, nature of the dyadic relationship, interaction with role strain and possibly self-efficacy in influencing overall burden, we argue that the information provided by WaP in enhancing our understanding of the caregiving burden phenomena would be too rich to ignore (3, 13, 50). In support of this, a recent study of the 12-item ZBI in Hong Kong Chinese dementia caregiver found Bédard’s two-factor model of personal strain and role strain to be inadequate, and that the best fit was obtained with a three-factor model that also included “self-criticism” comprising items 20 and 21 (10). Acknowledging the need for brevity in a clinical instrument, our approach may be the first to expand the number of items to better delineate the WaP construct before employing item-reduction strategies to only retain items that show good psychometric properties.

Some limitations are worth noting. While the cross-sectional design is adequate in validating the four-factor model in a fresh sample, novel findings such as the difference across CDR stages for different dyadic relationships need to be interpreted with caution and preferably replicated in longitudinal studies. The lack of comprehensive data across the spectrum of cognitive impairment in the sibling group also limits interpretability. In addition, the small *R*^2^ for the regression models suggests that there are other unaccounted-for factors that affect the variance of total ZBI and its dimensions in the models. For instance, information on socioeconomic factors such as financial status, whether one or more caregivers were involved and the degree of social support from other family caregivers, as well as variability in and access to healthcare services and community support, are potentially important factors that can mediate burden. Also, our study population of “middle-old” patients in a predominantly Chinese multiethnic population in an Asian country may limit the generalizability of our findings to the “oldest-old” (51) and other sociocultural context; nonetheless, the coherence with other studies across different cultures that demonstrated WaP to be a distinct factor supports that WaP is possibly a phenomenon that is applicable across different cultures. Finally, to retain the representativeness of our sample relative to the naturalistic multiethnic setting of Singapore, we elected to retain the Malay and Indian participants in our study. Similarly, to avoid selecting a skewed population of more highly educated English-speaking caregivers, we employed both English and Chinese versions of the ZBI so that the subset of non-English-speaking caregivers would not be excluded. We were mindful to utilize a rigorously validated version of the ZBI (30) and believe that this would also improve the external validity of our results in other multiethnic societies.

In conclusion, our study supports the findings of earlier studies that WaP is a distinct dimension of caregiving burden in addition to role and personal strain in the multidimensional of ZBI-defined burden. The four-factor structure that splits role strain into two factors yielded the best fit. WaP is predicted by NPI-severity and adult child relationship, but not NPI-distress or physical function. Understanding how WaP trends across the CDR stages for different dyadic relationships can fuel future research to explore the potential of WaP as a possible intervention target in the milder stages of burden if detected early. Moving forward, we therefore recommend an expansion of items in WaP and that future studies examine burden as a multidimensional construct beyond the total score to incorporate role strain, personal strain, and WaP. More research is also needed to understand the impact on caregiver burden of subjective presenting complaint and objective primary domain of cognitive involvement, and whether this can help to delineate subsets of WaP.

## Ethics Statement

As this study involved the retrospective review of medical records of patients attending the Memory clinic as part of a registered database, waiver of informed consent was approved by the Institutional Review Board of the National Healthcare Group.

## Author Contributions

RL was responsible for data analysis and writing the manuscript. WL supervised the data analysis and was involved in the critical appraisal of the manuscript. MC, PC, BT and NA were involved in study design and critical appraisal of the manuscript.

## Conflict of Interest Statement

The authors declare that the research was conducted in the absence of any commercial or financial relationships that could be construed as a potential conflict of interest.
